# Whole-Genome Sequence of a *Mycobacterium goodii* Isolate from a Pediatric Patient in South Africa

**DOI:** 10.1128/genomeA.01478-17

**Published:** 2018-01-18

**Authors:** Mushal Allam, Lavania Joseph, Farzana Ismail, Halima Said, Nazir A. Ismail, Arshad Ismail, Senzo Mtshali, Florah Mnyameni, Pierre Goussard, Jade C. Pekeur, Adré Lourens, Shaheed V. Omar

**Affiliations:** a Sequencing Core Facility, National Institute for Communicable Diseases, National Health Laboratory Service, Johannesburg, South Africa; b Centre for Tuberculosis, National Institute for Communicable Diseases, Johannesburg, South Africa; c Department of Medical Microbiology, University of Pretoria, Pretoria, South Africa; d Department of Medical Microbiology, University of the Free State, Bloemfontein, South Africa; e Department of Paediatrics and Child Health, Faculty of Health Sciences, Stellenbosch University, Cape Town, South Africa; f Division of Medical Microbiology, Department of Pathology, Stellenbosch University and National Health Laboratory Service, Cape Town, South Africa

## Abstract

We describe here the draft genome sequence of a *Mycobacterium goodii* isolate from a pediatric patient in Western Cape, South Africa. To our knowledge, this is the second reported genome of this rapidly growing nontuberculous mycobacterial species.

## GENOME ANNOUNCEMENT

Nine specimens from a pediatric patient were submitted for culture, including specimens obtained from bronchoalveolar lavage fluid, tissue, gastric washing fluid, and a pus swab for tuberculosis investigation. No acid-fast bacilli were observed on direct auramine staining and microscopy. Cultivation of mycobacteria was successful for the gastric washing fluid, pus swab, and brochoalveolar lavage fluid specimens within a median time of 4 days (range, 2 to 14 days) using the MGIT 960 instrument (BD, Sparks, MD, USA). The presence of noncorded acid-fast bacilli was observed when we performed Ziehl-Neelsen staining on these cultures. The tuberculosis antigen MPT64 rapid test (SD Bioline) for detection of the presence of *Mycobacterium tuberculosis* complex was negative. Thereafter, species identification was performed using the Genotype Mycobacterium CM version 2.0 (Hain Lifescience GH, Nehren, Germany) on an isolate obtained from a gastric washing fluid specimen which was identified as a *Mycobacterium* species. The isolate, which was designated strain ST0139456, was then subjected to 16S rRNA sequencing using the forward primer 5′-AGTTTGATCMTGGCTCAG-3′ and reverse primer 5′-GGACTACHAGGGTATCTAAT-3′, and the resulting BLAST search (https://blast.ncbi.nlm.nih.gov) confirmed 99% homology to *Mycobacterium goodii*, a nontuberculous mycobacterium species of the *Mycobacterium smegmatis* group. The species identification as *Mycobacterium goodii* was further confirmed using the GenoType Mycobacterium AS version 1.0 kit (Hain Lifescience GH) and whole-genome sequencing.

Paired-end libraries were prepared using the Nextera XT DNA library kit, followed by 2- × 300-bp sequencing on a MiSeq instrument (Illumina, San Diego, CA, USA). The sequenced reads were quality trimmed using Sickle version 1.33 (https://github.com/najoshi/sickle) and *de novo* assembled using SPAdes genome assembler version 3.5 (1). The assembly contains 156 contig sequences of longer than 200 bp and covers 6,621,508 bp, with a G+C content of 67.07% and an *N*_50_ of 116,855 bp. Genome annotation was performed via the NCBI Prokaryotic Genome Annotation Pipeline (PGAP) (2). The total number of 6,514 genes predicted by PGAP includes 6,281 protein-coding genes, 175 pseudogenes, and 58 RNA genes.

### Accession number(s).

The draft genome sequence has been deposited at NCBI under the BioProject number PRJNA415539, BioSample number SAMN07828250, and GenBank accession number PEBB00000000.
